# *Sporobolomyces lactucae* sp. nov. (Pucciniomycotina, Microbotryomycetes, Sporidiobolales): An Abundant Component of Romaine Lettuce Phylloplanes

**DOI:** 10.3390/jof8030302

**Published:** 2022-03-16

**Authors:** Samira Fatemi, Danny Haelewaters, Hector Urbina, Samuel Brown, Makenna L. Houston, M. Catherine Aime

**Affiliations:** 1 Department of Botany and Plant Pathology, Purdue University, West Lafayette, IN 47907, USA; sfatemi@purdue.edu (S.F.); danny.haelewaters@gmail.com (D.H.); hurbinay@gmail.com (H.U.); sambrown1119@gmail.com (S.B.); makennahouston@yahoo.com (M.L.H.); 2 Research Group Mycology, Department of Biology, Ghent University, 9000 Ghent, Belgium; 3 Faculty of Science, University of South Bohemia, 370 05 České Budějovice, Czech Republic; 4 Division of Plant Industry, Florida Department of Agriculture and Consumer Services, Gainesville, FL 32608, USA

**Keywords:** one new taxon, Basidiomycota, fungi, microbial ecology, multi-locus phylogeny, taxonomy, yeasts

## Abstract

Shifts in food microbiomes may impact the establishment of human pathogens, such as virulent lineages of *Escherichia coli*, and thus are important to investigate. Foods that are often consumed raw, such as lettuce, are particularly susceptible to such outbreaks. We have previously found that an undescribed *Sporobolomyces* yeast is an abundant component of the mycobiome of commercial romaine lettuce (*Lactuca sativa*). Here, we formally describe this species as *Sporobolomyces lactucae* sp. nov. (Pucciniomycotina, Microbotryomycetes, and Sporidiobolales). We isolated multiple strains of this yeast from commercial romaine lettuce purchased from supermarkets in Illinois and Indiana; additional isolates were obtained from various plant phylloplanes in California. *S. lactucae* is a red-pigmented species that is similar in appearance to other members of the genus *Sporobolomyces*. However, it can be differentiated by its ability to assimilate glucuronate and D-glucosamine. Gene genealogical concordance supports *S. lactucae* as a new species. The phylogenetic reconstruction of a four-locus dataset, comprising the internal transcribed spacer and large ribosomal subunit D1/D2 domain of the ribosomal RNA gene, translation elongation factor 1-α, and cytochrome B, places *S. lactucae* as a sister to the *S. roseus* clade. *Sporobolomyces lactucae* is one of the most common fungi in the lettuce microbiome.

## 1. Introduction

The genus *Sporobolomyces* was erected by Kluyver and van Niel (1924) [1] to accommodate asexual basidiomycetous yeasts, with *Sporobolomyces salmonicolor* as the type species [2,3]. Similar species typified by a sexual morph were placed in *Sporidiobolus* [3,4,5]. It is now known that yeasts previously placed in form genus *Sporobolomyces* can be found across the three subphyla of Basidiomycota [6,7] and even in Ascomycota [8]. *Sporobolomyces salmonicolor*, however, belongs to Sporidiobolales (Pucciniomycotina, Microbotryomycetes), and *Sporidiobolus* is now considered a synonym of *Sporobolomyces* [9]. Currently, 22 species of *Sporobolomyces* sensu stricto (s.s.) are accepted [3,10,11], although estimates are that the genus contains upwards of 60 species [12]. All known species in the genus produce an asexual morph and reproduce by ballistoconidia. Some species produce a sexual morph and pseudohyphae in addition to yeast cells [13].

Yeasts in the genus *Sporobolomyces* are known for their bright red, orange, or pink appearance in culture [2] and have been studied for a variety of applications. The red pigmentation of the yeasts is due to the production of carotenoids such as beta-carotene [14,15]. The carotenoid-producing capability of *Sporobolomyces* yeasts has been of interest to the field of biotechnology to develop commodities such as pigments [14,15,16]. *Sporobolomyces roseus* exhibits antimicrobial activity inhibiting the growth of *Pseudomonas fluorescens* and *Staphylococcus aureus*, two bacteria that are both known to infect humans as opportunistic pathogens [17]. As a biological control agent, *S. roseus* has been effective against *Cochliobolus sativus* (common root rot) [18].

*Sporobolomyces* yeasts grow in various habitats, such as aquatic systems, soil, and plant phylloplanes [2,7,10,12]. They are best known for their association with plant leaves, with many organisms from the genus first isolated from the phylloplane [12,19]. *Sporobolomyces* yeasts are cosmopolitan in this regard and are capable of growing on a wide variety of plants, including agricultural crops [12,20,21,22,23,24]. The yeasts of Sporidiobolales are known to inhabit vegetable surfaces with little, if any, association with food spoilage [25,26].

We have previously found that the yeasts of Sporidiobolales are represented in the phylloplane of commercially grown romaine lettuce [12], which is consistent with a previous study on the lettuce microbiome [23]. We then characterized the mycobiome (fungi of the microbiome) of romaine lettuce obtained in Urbina and Aime [12]. By conducting this characterization, we found that over 25% of the mycobiome is represented by a single *Sporobolomyces* species, which is previously undescribed [24]. Although basidiomycetous yeasts comprise a small fraction of the romaine lettuce phylloplane microbiome, Sporidiobolales yeasts are the most common fungi present [12] and are represented overwhelmingly by *Sporobolomyces* spp. [24]. Here, we describe the most abundant of these red yeasts, *Sporobolomyces lactucae* sp. nov., and discuss its ecology and natural range.

## 2. Materials and Methods

### 2.1. Lettuce Leaf Preparation and Culturing of Fungal Isolates

Lettuce leaf homogenization and culture plating were completed as described in previous work [12,24]. In brief, commercial lettuce was purchased from grocery stores in Illinois and Indiana. Lettuce leaves were homogenized in 225 mL of 100 µM phosphate buffer (5.4 g monosodium phosphate L^−1^ and 8.7 g disodium phosphate L^−1^). Aliquots were plated on yeast extract–peptone–glucose agar with 25 µg chloramphenicol mL^−1^ and 50 µg ampicillin mL^−1^ to inhibit bacterial growth (BD, Franklin Lakes, NJ, USA; Thermo Fisher, Waltham, MA, USA). Isolates collected in California were obtained by using the ballistospore drop method [27]. Sections from leaves collected in the field were secured with petroleum jelly to the inside of a Petri plate lid. Ballistospores that dropped from the leaf-inhabiting yeasts grew on either potato dextrose agar (PDA) or yeast malt agar (YMA) (BD; Thermo Fisher). Subculturing was repeated until axenic cultures were obtained and maintained on PDA. Back-up cultures for long-term preservation were prepared in 40% (*v*/*v*) glycerol for −80 °C storage and on PDA slants for 4 °C storage. Working cultures were incubated on PDA at room temperature (25 °C). Live cultures were deposited in the Agricultural Research Service Culture Collection (NRRL) and the Westerdijk Fungal Biodiversity Institute (CBS). All isolates obtained for this study and their origins are presented in Supplementary Table S1.

### 2.2. Morphological and Physiological Characterization

The morphological and physiological description was performed according to Suh et al. [28]; their protocols conform to the standard outlined in *The Yeasts* [29]. Culture morphology was observed on YMA, corn meal agar (CMA), and YM broth (BD; Thermo Fisher). After seven-day incubation, colonies were described in terms of elevation, margin, color (oac; [30]), form, and surface texture. Individual cells were examined under a compound microscope (Olympus BH-2; Tokyo, Japan). Micrographs were captured using an Olympus SC30 camera. A total of 30–180 cells were measured per isolate per treatment using Piximètre v5.10 (http://www.piximetre.fr/, accessed on 4 May 2021). Carbon and nitrogen assimilations were performed according to Suh et al. [28]. Positive assimilations marked with “+++” or “++” were reassigned as “+” while negative assimilations were assigned as “−”; weak growth was denoted as “(w)”, and delayed growth was denoted as “(d)”. This modified scale was used to normalize our data and allow for comparison across previously published data with varying scales.

### 2.3. DNA Extraction, PCR Amplification, and Sequencing

DNA was extracted using the Wizard Genomic DNA Purification Kit (Promega, Madison, WI, USA) following the manufacturer’s instructions. Alternatively, PCR amplifications were performed directly from colonies [27]. Various loci were sequenced to perform phylogenetic analyses as in Urbina and Aime [12]. The internal transcribed spacer (ITS) region was amplified using ITS1F and ITS4 primers [31,32]; the small ribosomal subunit (SSU) ribosomal RNA gene (rDNA) using NS1 and NS4 [31]; large ribosomal subunit (LSU) rDNA using LR0R and LR6 [33,34]; translation elongation factor 1-α (*tef1*) using EF1-983F and EF1-1567R [35]; and cytochrome B (*cytb*) using E1M4 and E2mr3 [36].

PCR protocols followed Toome et al. [37] for SSU, ITS, and LSU. For *tef1*, we used a touchdown PCR protocol as in Wang et al. [38]. For *cytb*, we followed Wang and Bai [39]. Purification and Sanger sequencing with amplification primers were outsourced to Gene-wiz, Inc. (South Plainfield, NJ, USA). Raw sequence reads were assembled and edited in Sequencher v. 5.2.3 (Gene Codes, Ann Arbor, MI, USA). Newly generated sequences were submitted to NCBI GenBank (accession numbers in Supplemental Table S1).

### 2.4. Phylogenetic Inferences and Species Concepts

Generated sequences of each locus were blasted against the NCBI GenBank standard *nr*/*nt* nucleotide database (http://ncbi.nlm.nih.gov/blast/Blast.cgi, accessed on 27 January 2022) to confirm identity [40]. For phylogenetic placement of our isolates, we downloaded ITS, LSU, *tef1*, and *cytb* ex-type sequences of *Sporobolomyces* from GenBank, following Urbina and Aime [12], Li et al. [11], and Tan et al. [41] as a guide for taxon selection to represent the 22 currently accepted species (Table 1). *Rhodosporidiobolus microsporus* and *Rhodotorula babjevae* were selected as outgroup taxa [12]. Sequences were aligned using MUSCLE v.3.8.1551 via the CIPRES Science Gateway [42,43]. The newly created multiple sequence alignment for each dataset was trimmed using TrimAI v.1.2.59 via CIPRES [43,44]. After trimming, single-locus trees were constructed using the Random Axelerated Maximum Likelihood (RAxML) v.8 program [45] available through CIPRES [43].

We applied a genealogical gene concordance hypothesis for species delimitation [54] by using information from multiple loci to establish overlapping phylogenies that are in consensus. Single-locus trees were constructed as above. Concordance was determined by hand. The SSU region was not informative and, thus, was not used in the multi-locus phylogenetic reconstruction.

The *tef1* gene, *cytb* gene, LSU D1/D2 domain (28S rDNA region), and the ITS region were used for the construction of a four-locus phylogeny. The ITS and LSU D1/D2 domain regions are used here because they are widely used barcode regions for fungi, with LSU particularly suitable for yeasts. The genes *tef1* and *cytb* were selected to represent nuclear and mitochondrial protein coding regions, respectively. The individual datasets were concatenated by using Mesquite v.3.61 [55]. Appropriate models for nucleotide substitution were selected using ModelFinder v1.6.12 [56] considering the Akaike Information Criterion. The models selected were TIM2 + F + R3 for ITS (−lnL = 2883.946), GTR + F + R2 for LSU (−lnL = 2077.917), GTR + F + R3 for *tef1* (−lnL = 2235.566), and TVM + F + I + G4 for *cytb* (−lnL = 1704.151). Maximum likelihood was performed with IQ-TREE v.1.6.12 [57] under partitioned models [58]. Ultrafast bootstrapping was performed with 1000 replicates [59]. The reconstruction was visualized in FigTree v1.4.4 (http://tree.bio.ed.ac.uk/software/figtree/, accessed on 27 January 2022).

### 2.5. Environmental Determination of S. lactucae

To infer the broader distribution and habitats of *S. lactucae,* a second dataset was constructed of ITS sequences. The holotype strain of *S. lactucae* sp. nov. was pairwise aligned with environmental sequences deposited in GenBank that shared ≥98.0% identity. Environmental sequences were edited, aligned, and trimmed as above. We reconstructed an ITS-based phylogeny of the environmental sequences via IQ-TREE and inferred matching *S. lactucae* isolates based on this phylogeny. The final environmental dataset included 118 ITS sequences, 19 of which are *Sporobolomyces* s.s. type species, and was evaluated by using the K3Pu + F + G4 model (−lnL = 2533.283).

## 3. Results

### 3.1. Phylogenetic Analyses

By performing gene genealogical concordance tests, each single-locus phylogenetic tree supported the delineation of *S. lactucae* as a new species. A congruence test of the four loci (ITS, LSU, *tef1*, and *cytb*) showed similar results with respect to our isolates. These single-locus phylogenies can be found in Supplemental Figures S1–S4. After determining concordance, a multi-locus phylogeny was constructed.

**Figure 1:**
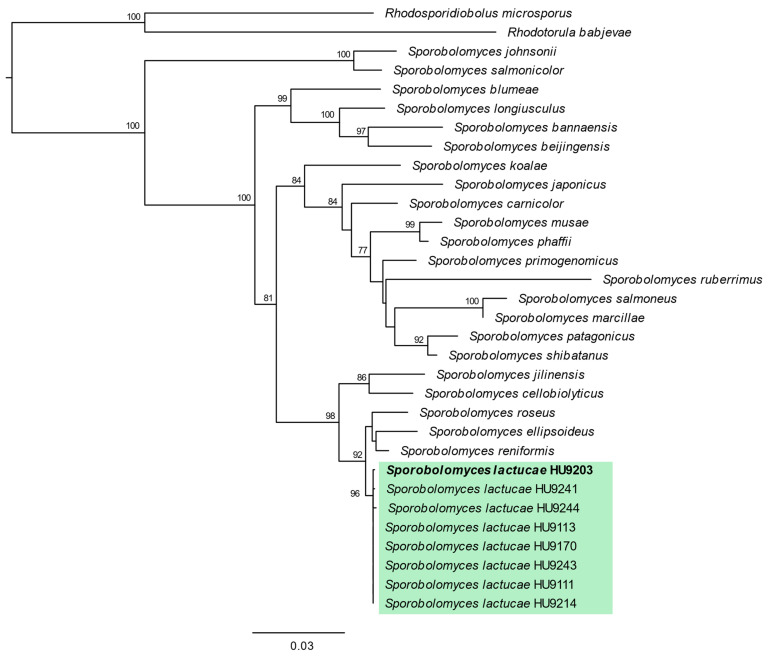
Phylogeny of *Sporobolomyces* s.s. reconstructed from a four-locus dataset. Only ex-type strains were included in this analysis, and the holotype selected for *S. lactucae* sp. nov. is highlighted in bold. Threshold for maximum likelihood bootstrap values was 70.

The final four-locus dataset (Figure 1) consisted of 32 taxa that are accepted as *Sporobolomyces* s.s.: 31 with ITS sequence data, 31 with LSU, 30 with *tef1*, and 28 with *cytb*. For ITS sequences, 610 characters constituted the dataset, of which 121 were parsimony-informative and 423 were constant. The LSU dataset consisted of 613 characters, with 85 parsimony-informative and 477 constant characters. The *tef1* dataset comprised 473 characters: 85 were parsimony-informative and 359 were constant. The *cytb* dataset contained 414 characters, of which 60 were parsimony-informative and 293 were constant. Eight isolates form a well-supported, monophyletic lineage representative of *S. lactucae*: HU9111, HU9113, HU9170, HU9203, HU9214, HU9241, HU9243, and HU9244 (Figure 1).

**Figure 2:**
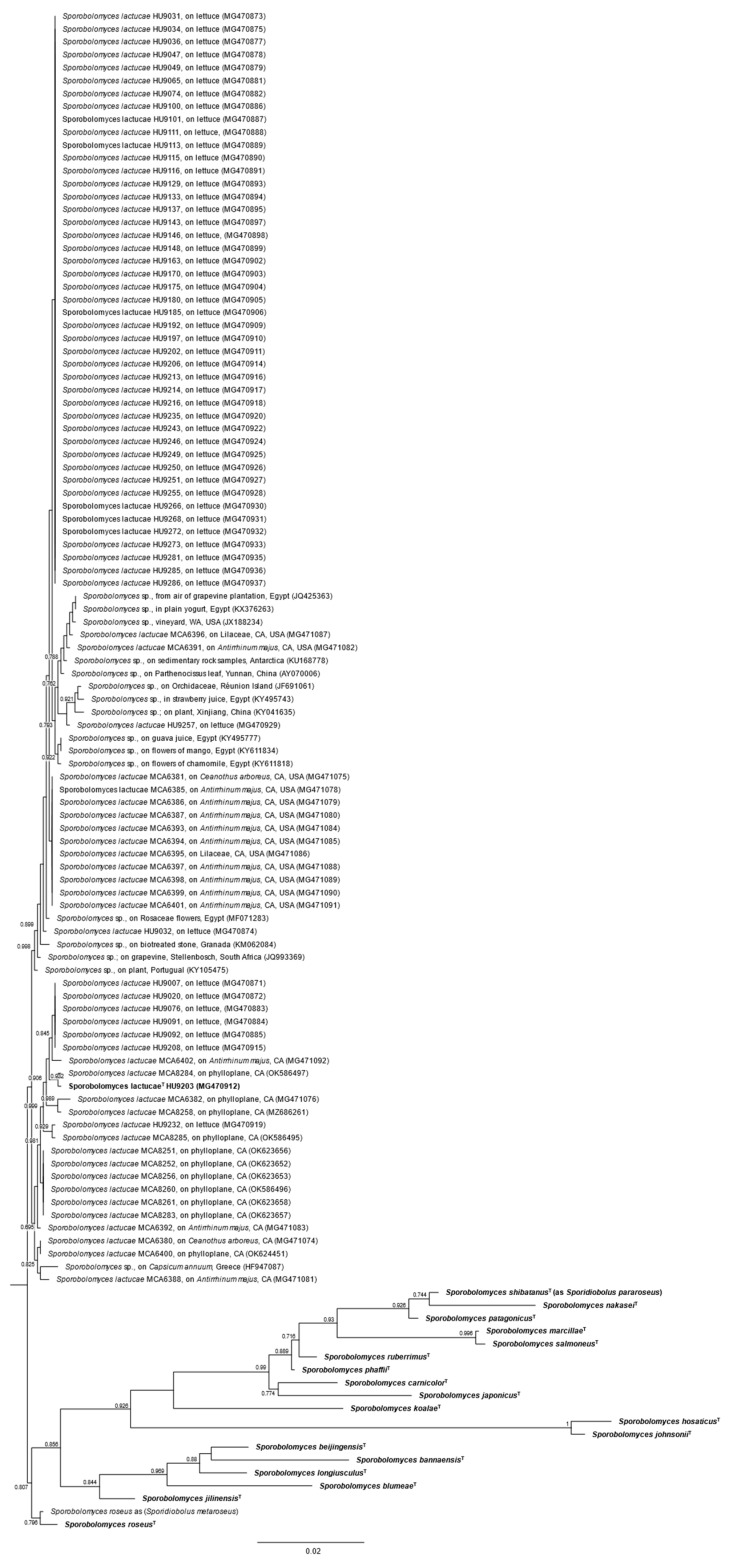
Phylogeny of environmental *Sporobolomyces lactucae* sequences reconstructed from ITS dataset. *Sporobolomyces* s.s. ex-type sequences are indicated with ^T^.

In total, we recovered 66 *S. lactucae* isolates from commercial romaine lettuce. Additionally, we recovered 27 *S. lactucae* isolates from the phylloplanes of Rhamnaceae (*Ceanothus arboreus*), Plantaginaceae (*Antirrhinum majus*), and Lilaceae in two different years, all within the San Francisco Bay Area region of California (Figure 2; Supplemental Table S1). To better infer true *S. lactucae* environmental sequences, we reconstructed an ITS-based phylogeny. For our environmental dataset, we found 99 *Sporobolomyces* ITS sequences from ten studies or surveys (including the present study) that shared at least 98% sequence identity with *S. lactucae*.

### 3.2. Taxonomy

**Figure 3:**
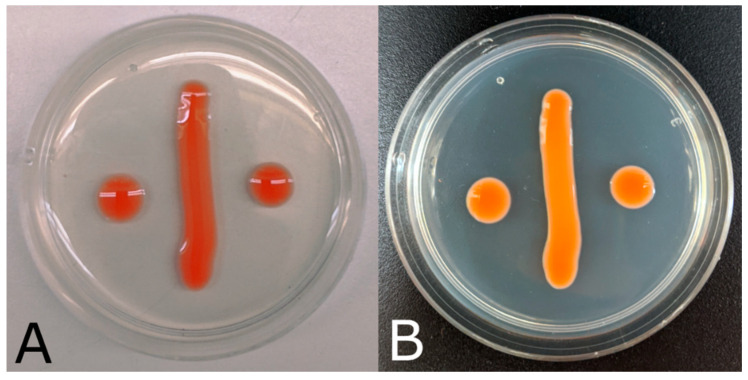
*Sporobolomyces lactucae*. (**A**) Morphology of *S. lactucae* after 7-day incubation at ambient conditions on PDA, photographed against a white background. (**B**) The same plate photographed against a black background.

**Figure 4:**
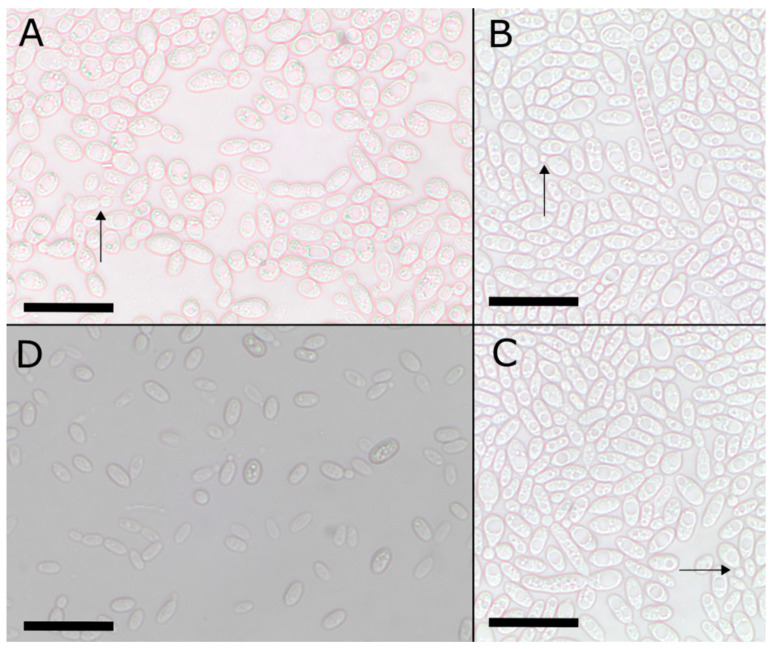
*Sporobolomyces lactucae*, micromorphological characteristics. Scale bar = 20 µm. **Clockwise from top left:** (**A**–**C**) Cells of *S. lactucae* at 400× magnification, incubated for 7 days on CMA as a Dalmau culture at ambient conditions (25 °C). Ballistoconidia arising from sterigmata are denoted with arrows. (**D**) Cells of *S. lactucae* grown in YM broth for 7 days in ambient conditions (25 °C). Cells were mounted on 2% potassium hydroxide for microscopy.

***Sporobolomyces lactucae***, Fatemi, Urbina & Aime, **sp. nov.**, MycoBank MB 840687. Figure 3 and Figure 4. Ex-holotype identifiers: CBS 16795; NRRL Y-64010.

Etymology: *lactucae* (Latin), referring to the genus of the lettuce plant from which the holotype isolate was sourced.

Diagnosis: Similar to *S. jilinensis* and *S. roseus* but differing in the ability to assimilate glucoronate and D-glucosamine but not lactate or citrate.

Typification: USA, Illinois, Urbana-Champaign, from leaves of commercial *Lactuca sativa* (Asterales, Asteraceae), 9 May 2016, H. Urbina HU9203 (**holotype** PUL F27743 preserved as dried inert culture). Isotype PUL F27744 preserved as dried inert culture. Ex-holotype cultures at CBS (CBS 16795) and NRRL (Y-64010). Ex-holotype GenBank accession numbers: MG588994 (SSU); MG470912 (ITS); MG588947 (LSU); MG589082 (*tef1*); MG589041 (*cytb*).

Habitat and distribution: on leaf surfaces, particularly those of agricultural products, in mild or Mediterranean climates.

Description: In the asexual state, colonies are orange-pink in color (oac616) after 7 d incubation at 25 °C on PDA and YMA. Colonies are smooth and glistening, varying between circular and irregular with the entire margin. Colonies are raised in elevation. After 7 d incubation in YM broth, single cells appeared ellipsoidal, 5–11 µm × 3–5 µm, and uninucleate. On CMA, cells measured 5–10 µm × 3–6 µm. A single large vacuole forms in the cells. The formation of ballistoconidia was observed on CMA; new cells arise from sterigmata on mother cells. Pseudohyphae were not observed, but small chains of cells (usually about three cells) were rarely observed. No sexual stage was observed.

Fermentation of glucose was negative. Growth was observed on media containing yeast extract (1% *w*/*v*) and agar (2% *w*/*v*) with the following supplements: 50% *w*/*v* glucose or 60% *w*/*v* glucose. Negligible/weak growth was seen on the same media supplemented with 10% *w*/*v* sodium chloride and 16% *w*/*v* sodium chloride. Assimilation was positive for the following carbon compounds: glucose, galactose (weak, delayed), sucrose, maltose, cellobiose (weak, delayed), soluble starch (weak), glucono-1,5-lactone (weak), glucuronate, galacturonic acid (weak), ethanol (weak), and propane-1,2-diol (weak). Assimilation was negative for the carbon compounds lactose, inulin, myo-inositol, lactate, citrate, and methanol. Assimilation was positive for the nitrogen compounds potassium nitrate, sodium nitrate, ethylamine (weak), L-lysine, D-glucosamine, creatine (weak), creatinine (weak), and D-tryptophan (weak). Assimilation was negative for the nitrogen compound imidazole.

Additional materials: USA. INDIANA: Lafayette, commercial lettuce leaf, 30 April 2016, H. Urbina HU9007; 30 April 2016, H. Urbina HU9020; 30 April 2016, H. Urbina HU9031; 30 April 2016, H. Urbina HU9034; 30 April 2016, H. Urbina HU9035; 30 April 2016, H. Urbina HU9036; 2 May 2016, H. Urbina HU9047; 2 May 2016, H. Urbina HU9049; 6 May 2016, H. Urbina HU9062; 6 May 2016, H. Urbina HU9065; 6 May 2016, H. Urbina HU9074; 6 May 2016, H. Urbina HU9076; 2 May 2016, H. Urbina HU9091; 6 May 2016, H. Urbina HU9092; 6 May 2016, H. Urbina HU9100; 6 May 2016, H. Urbina HU9101; 2 May 2016, H. Urbina HU9111; 6 May 2016, H. Urbina HU9113; 6 May 2016, H. Urbina HU9115; 6 May 2016, H. Urbina HU9116; 6 May 2016, H. Urbina HU9128; 6 May 2016, H. Urbina HU9129; 6 May 2016, H. Urbina HU9133; 6 May 2016, H. Urbina HU9137; 6 May 2016, H. Urbina HU9143; 6 May 2016, H. Urbina HU9146; 6 May 2016, H. Urbina HU9148. USA. INDIANA: West Lafayette, commercial lettuce leaf, 8 May 2016, H. Urbina HU9152; 8 May 2016, H. Urbina HU9155; 8 May 2016, H. Urbina HU9163; 8 May 2016, H. Urbina HU9170; 8 May 2016, H. Urbina HU9175. USA. ILLINOIS: Urbana-Champaign, commercial lettuce leaf, 9 May 2016, H. Urbina HU9180; 9 May 2016, H. Urbina HU9185; 9 May 2016, H. Urbina HU9188; 9 May 2016, H. Urbina HU9192; 9 May 2016, H. Urbina HU9197; 9 May 2016, H. Urbina HU9202; 9 May 2016, H. Urbina HU9206; 9 May 2016, H. Urbina HU9208; 9 May 2016, H. Urbina HU9213; 9 May 2016, H. Urbina HU9214; 9 May 2016, H. Urbina HU9216; 9 May 2016, H. Urbina HU9233; 9 May 2016, H. Urbina HU9235. USA. ILLINOIS: Chicago, commercial lettuce leaf, 19 May 2016, H. Urbina HU9241; 19 May 2016, H. Urbina HU9243; 19 May 2016, H. Urbina HU9249; 19 May 2016, H. Urbina HU9250; 19 May 2016, H. Urbina HU9251; 19 May 2016, H. Urbina HU9255; 19 May 2016, H. Urbina HU9257; 19 May 2016, H. Urbina HU9266; 19 May 2016, H. Urbina HU9268; 19 May 2016, H. Urbina HU9272; 19 May 2016, H. Urbina HU9273; 19 May 2016, H. Urbina HU9279; 19 May 2016, H. Urbina HU9281; 19 May 2016, H. Urbina HU9285; 19 May 2016, H. Urbina HU9286. USA. CALIFORNIA: Berkeley, diseased leaf of *Ceanothus arboreus* (Rosales, Rhamnaceae), 5 August 2016, M.C. Aime MCA6380; 5 August 2016, MCA6381; healthy leaf of *C. arboreus*, 5 August 2016, M.C. Aime MCA6382; healthy leaf of *Antirrhinum majus* (Lamiales, Plantaginaceae), 5 August 2016, M.C. Aime MCA6385; diseased leaf of *A. majus*, 5 August 2016, M.C. Aime MCA6386; 5 August 2016, M.C. Aime MCA6387; healthy flower of *A. majus*, 5 August 2016, M.C. Aime MCA6391; 5 August 2016, M.C. Aime MCA6392; 5 August 2016, M.C. Aime MCA6393; 05 Aug 2016, M.C. Aime MCA6394; necrotic leaf (Liliaceae), 5 August 2016, M.C. Aime MCA6395; 5 August 2016, M.C. Aime MCA6396; diseased leaf of *A. majus*, 5 August 2016, M.C. Aime MCA6397; necrotic leaf of *A. majus*, 5 August 2016, M.C. Aime MCA6398; diseased leaf of *A. majus*, 5 August 2016, M.C. Aime MCA6399; phylloplane of undetermined host, 5 August 2016, M.C. Aime MCA6400; necrotic leaf of *A. majus*, 5 August 2016, M.C. Aime MCA6401; healthy flower of *A. majus*, 5 August 2016, M.C. Aime, MCA6402; Santa Cruz, phylloplane of undetermined host, 8 March 2019, M.C. Aime, MCA8251; phylloplane of undetermined host, 8 March 2019, M.C. Aime, MCA8252; phylloplane of undetermined host, 8 March 2019, M.C. Aime, MCA8256; phylloplane of undetermined host, 8 March 2019, M.C. Aime, MCA8258; phylloplane of undetermined host, 8 March 2019, M.C. Aime, MCA8260; phylloplane of undetermined host, 8 March 2019, M.C. Aime, MCA8261; phylloplane of undetermined host, 8 March 2019, M.C. Aime, MCA8283; phylloplane of undetermined host, 8 March 2019, M.C. Aime, MCA8284; phylloplane of undetermined host, 8 March 2019, M.C. Aime, MCA8285.

Notes: Because of the high similarity in colony morphology, colony color, and individual cell shape between yeasts of *Sporobolomyces*, assimilations are a more effective means of diagnosis. *S. lactucae* is weak in its assimilation of ethanol, but *S. jilinensis* and *S. roseus* more readily assimilate the compound [5,13,21]. Glucuronate and D-glucosamine are both substrates that *S. roseus* is unable to assimilate, while *S. lactucae* can. *S. jilinensis* is also unable to assimilate D-glucosamine (Table 2). Assimilations of galactose, cellobiose, soluble starch, and ethanol are all weak in *S. lactucae*, while the closely related species *S. roseus* can assimilate all four compounds (Table 2). *S. lactucae* is also capable of growth on high osmotic media (50% *w*/*v* glucose) while *S. roseus* experiences variable growth and *S. jilinensis* experiences no growth on the same type of media. Most *S. lactucae* cultures showed visible growth within one to two days of incubation, with full colony growth displayed by day seven (Figure 3).

## 4. Discussion

*Sporobolomyces lactucae* was the most frequently isolated yeast from phylloplanes of commercial romaine lettuce purchased from grocery stores in Illinois and Indiana, USA [24]. Of the thousands of phylloplane isolates of Sporidiobolales we have made throughout the world ([12]; M.C. Aime, unpubl.), we have recovered *S. lactucae* only from plant samples in the San Francisco and Monterrey Bay areas of California (Supplemental Table S2); identical sequences have been detected by others in areas across the world that have similar climates (Table 3). The majority of lettuce produced in the USA is grown in California [60], and we were able to trace the romaine lettuce origins for most of our samples to the Salinas Valley in California (Supplemental Table S3). It is well-documented that pathogenic microorganisms can spread through the production, distribution, and preparation of food, with greater risks of foodborne illnesses for foods consumed raw such as lettuce [61]. It stands to reason that commensal organisms are moved through our food distribution systems as well. In this case, *S. lactucae* isolated from lettuce purchased in the Midwest may very well have originated in California. The ability to trace commensal microbial organisms through food distribution may improve our ability to trace pathogenic outbreaks.

Much like other members of Sporidiobolales, *S. lactucae* appears to be widespread, occupying various habitats [12]. We found sequences consistent with *S. lactucae* in public databases from regions that experience a Mediterranean climate (Table 3). Half of the environmental samples were isolated in Egypt. The other half are distributed across Réunion Island (Indian Ocean), South Africa, Greece, Portugal, Granada, and Pullman (Washington, USA). Additionally, our environmental analysis indicates the potential presence of *S. lactucae* in Antarctica as well as Yunnan Province, China. Although the substrate from which environmental sequences were obtained vary, most of these originated from agricultural samples. Substrates were either agricultural crops, such as grape berries; in food products such as strawberry or guava juice; or flowers of agricultural or horticultural plants.

## Figures and Tables

**Table 1:** Isolates used in the phylogenetic reconstruction of *Sporobolomyces* s.s. Four loci were selected in this analysis: *cytb*, *tef1*, LSU D1/D2 domain, and ITS. Type strains are designated with ^T^.

Genus	Species	Authority	Strain	*tef1*	LSU	ITS	*cytb*	Source
*Rhodosporidiobolus*	*microsporus*	(Higham ex Fell, Blatt, and Statzell) Q.M. Wang, F.Y. Bai, M. Groenew., and Boekhout 2015	CBS 7041 ^T^	KJ707817	NG_042344	NR_073290	KJ707724	[7,46]
*Rhodotorula*	*babjevae*	(Golubev) Q.M. Wang, F.Y. Bai, M. Groenew., and Boekhout 2015	CBS 7808 ^T^	–	NG_042339	NR_077096	–	[46]
*Sporobolomyces*	*bannaensis*	F.Y. Bai and J.H. Zhao 2003	CBS 9204 ^T^	KJ707934	NG_068721	NR_073345	KJ707581	[7,46]
*Sporobolomyces*	*beijingensis*	F.Y. Bai and Q.M. Wang 2004	CGMCC 2.2365 ^T^	KJ707919	AY364837	NR_137663	KJ707588	[7,21]
*Sporobolomyces*	*blumeae*	M. Takash. and Nakase 2000	CBS 9094 ^T^	KJ707926	KY109742	NR_137641	KJ707673	[7,47]
*Sporobolomyces*	*carnicolor*	Yamasaki and H. Fujii ex F.Y. Bai and Boekhout	CBS 4215 ^T^	KJ707912	NG_067316	NR_137659	KJ707707	[7,48]
*Sporobolomyces*	*cellobiolyticus*	Q.M. Wang, F.Y. Bai and A.H. Li (2020)	CGMCC 2.5675 ^T^	MK849110	MK050406	MK050406	MK848982	[11]
*Sporobolomyces*	*ellipsoideus*	Q.M. Wang, F.Y. Bai and A.H. Li (2020)	CGMCC 2.5619 ^T^	MK849088	MK050409	MK050409	MK848957	[11]
*Sporobolomyces*	*japonicus*	Iizuka and Goto 1965	CBS 5744 ^T^	KJ707932	KY109745	NR_155844	KJ707578	[7,49]
*Sporobolomyces*	*jilinensis*	F.Y. Bai and Q.M. Wang 2004	CGMCC 2.2301 ^T^	KJ707913	NG_068244	NR_137664	KJ707583	[21]
*Sporobolomyces*	*johnsonii*	(Nyland) Q.M. Wang, F.Y. Bai, M. Groenew., and Boekhout 2015	CBS 5470 ^T^	KJ707931	NG_042343	NR_077090	–	[7,50]
*Sporobolomyces*	*koalae*	Satoh and Makimura 2008	CBS 10914 ^T^	KJ707850	NG_067317	NR_137556	KJ707604	[7,51]
*Sporobolomyces*	*lactucae*	–	HU 9214	MG589084	MG588949	MG470917	MG589043	[12]
*Sporobolomyces*	*lactucae*	Fatemi, Urbina, Haelew., and Aime 2022	HU 9203 ^T^	MG589082	MG588947	MG470912	MG589041	[12]
*Sporobolomyces*	*lactucae*	–	HU 9170	MG589079	MG588944	MG470903	MG589039	[12]
*Sporobolomyces*	*lactucae*	–	HU 9113	MG589077	MG588942	MG470889	MG589037	[12]
*Sporobolomyces*	*lactucae*	–	HU 9241	MG589086	MG588951	MG470921	MG589045	[12]
*Sporobolomyces*	*lactucae*	–	HU 9243	MG589087	MG588952	MG470922	MG589046	[12]
*Sporobolomyces*	*lactucae*	–	HU 9111	MG589076	MG588941	MG470888	MG589036	[12]
*Sporobolomyces*	*lactucae*	–	HU 9244	MG589088	MG588953	MG470923	MG589047	[12]
*Sporobolomyces*	*longiusculus*	(Libkind, Van Broock, and J.P. Samp.) Q.M. Wang, F.Y. Bai, M. Groenew., and Boekhout 2015	PYCC 5818 ^T^	KJ707929	NG_068720	NR_155773	KJ707668	[7,52]
*Sporobolomyces*	*marcillae*	Santa María 1958	JCM 6883 ^T^	KJ707933	–	–	KJ707725	[53]
*Sporobolomyces*	*musae*	Y.P. Tan, Marney, and R.G. Shivas 2021	BRIP 28276 ^T^	–	OK483137	OK483138	–	[41]
*Sporobolomyces*	*patagonicus*	Libkind, Van Broock, and J.P. Samp. 2005	CBS 9657 ^T^	KJ707928	KY109759	NR_137666	KP216520	[7,52]
*Sporobolomyces*	*phaffii*	F.Y. Bai, M. Takash., and Nakase 2002	CGMCC 2.2137 ^T^	KJ707918	NG_068245	NR_137660	KJ707577	[7,48]
*Sporobolomyces*	*primogenomicus*	Q.M. Wang and F.Y. Bai (2020)	JCM 8242 ^T^	MK848998	MK050417	MK050417	MK848872	[11]
*Sporobolomyces*	*reniformis*	Q.M. Wang, F.Y. Bai, and A.H. Li (2020)	CGMCC 2.5627 ^T^	MK849096	MK050408	MK050408	MK848965	[11]
*Sporobolomyces*	*roseus*	Kluyver and C.B. Niel 1924	CBS 486 ^T^	HM014022	NG_069417	NR_155845	KJ707569	D.A. Henk unpubl.; [7,49]
*Sporobolomyces*	*ruberrimus*	Yamasaki and H. Fujii ex Fell, Pinel, Scorzetti, Statzell, and Yarrow 2002	CBS 7500 ^T^	HM014017	NG_067252	NR_136959	KJ707643	D.A. Henk unpubl.; [50]
*Sporobolomyces*	*salmoneus*	Derx 1930	CGMCC 2.2195 ^T^	KJ707920	KY109767	KY105530	KJ707580	[7]
*Sporobolomyces*	*salmonicolor*	(B. Fisch. and Brebeck) Kluyver and C.B. Niel 1924	JCM 1841 ^T^	KJ707923	NG_056268	NR_149325	KJ707701	[7]
*Sporobolomyces*	*shibatanus*	(Okun.) Verona and Cif.	CBS 491 ^T^	HM014019	NG_067256	NR_155770	–	D.A. Henk unpubl.; [50]

**Table 2:** Assimilation and physiological results of *S. lactucae* and closely related species *S. jilinensis* and *S. roseus*. Weak assimilation is denoted with “w”; delayed growth with “d”; and variable growth with “v”.

	*Species*	*S. lactucae*	*S. jilinensis*	*S. roseus*
	Type Strain	HU 9203 (CBS 16795)	CGMCC 2.2301	CBS 486
	Reference	This Paper	[21]	[13]
*CARBON ASSIMILATIONS*	Glucose	+	+	+
	Galactose	+ (w, d)	+	+
	Sucrose	+	+	+
	Maltose	+	+	+
	Cellobiose	+ (w, d)	-	+
	Lactose	-	-	-
	Inulin	-	-	-
	Soluble starch	+ (w)	+	+
	myo-Inositol	-	-	-
	Glucono-1,5-lactone	+ (w)	n/a	n/a
	Glucuronate	+	n/a	-
	Galacturonic Acid	(w)	n/a	-
	Lactate	-	-(as DL-lactic acid)	+
	Citrate	-	-(as citric acid)	+
	Methanol	-	-	-
	Ethanol	(w)	+	+
*NITROGEN ASSIMILATIONS*	Propane-1,2-diol	(w)	n/a	n/a
	K Nitrate	+	+	+
	Na Nitrite	+	+	+
	Ethylamine	(w)	-	n/a
	L-lysine	+	+	n/a
	Cadaverine	(w)	+	n/a
	Creatine	(w)	n/a	n/a
	Creatinine	(w)	n/a	n/a
	D-glucosamine	+	-	-
	Imidazole	-	n/a	n/a
	D-tryptophan	(w)	n/a	n/a
*OTHER*	10% (*w*/*v*) NaCl	(w)	n/a	n/a
	16% (*w*/*v*) NaCl	(w)	n/a	n/a
	50% (*w*/*v*) Glucose	+	-	v
	60% (*w*/*v*) Glucose	+	n/a	n/a

**Table 3:** Environmental sequences of *S. lactucae* included in ecological determination. ITS sequences were aligned and trimmed; a phylogenetic reconstruction of the ITS sequences was made.

Accession Number	Genbank Identification	Strain Identification	Our Identification	Percent Identification	Locality	Substrate	Reference
AY070006	*Sporobolomyces* sp.	AS 2.2108	*Sporobolomyces lactucae*	99.82%	Yunnan, China	wilting leaf of Parthenocissus sp.	[48]
HF947090	*Sporobolomyces* sp. (as *Sporidiobolus* sp.)	–	*Sporobolomyces lactucae*	99.65%	Greece	phylloplane of Capsicum annuum	[62]
JF691061	Atractiellales	–	*Sporobolomyces lactucae*	99.46%	Réunion Island	orchid roots	[63]
JQ425363	*Sporobolomyces* sp. (as *Sporidiobolus* sp.)	JPS-2007a	*Sporobolomyces lactucae*	99.65%	Egypt	air, grapevine plantation	Z.S.M. Soliman unpubl.
JQ993369	*Sporobolomyces roseus*	IWBT-Y808	*Sporobolomyces lactucae*	99.47%	South Africa	wine grape berries	[64]
JX188234	*Sporobolomyces* sp. (as *Sporidiobolus* sp.)	JPS-2007a	*Sporobolomyces lactucae*	99.82%	Pullman, WA, USA	on Vitis vinifera	[65]
KM062084	*Sporobolomyces* sp.	2H-7	*Sporobolomyces lactucae*	99.65%	Granada	stone (biotreated)	[66]
KU168778	*Sporobolomyces roseus* (as *Sporidiobolus metaroseus*)	T11-22	*Sporobolomyces lactucae*	99.82%	Antarctica	rock	S. Barahona et al. unpubl.
KX376263	*Sporobolomyces roseus* (as *Sporidiobolus metaroseus*)	AUMC 10722	*Sporobolomyces lactucae*	99.65%	Egypt	yogurt	Z.S.M. Soliman unpubl.
KY105475	*Sporobolomyces roseus* (as *Sporidiobolus metaroseus*)	CBS 10225	*Sporobolomyces lactucae*	99.82%	Portugal	plant	[49]
KY495743	*Sporobolomyces roseus*	AUMC 10775	*Sporobolomyces lactucae*	99.12%	Egypt	strawberry juice	Z.S.M. Soliman unpubl.
KY495777	*Sporobolomyces roseus*	AUMC 11209	*Sporobolomyces lactucae*	99.82%	Egypt	guava juice	Z.S.M. Soliman unpubl.
KY611818	*Sporobolomyces roseus*	AUMC 11213	*Sporobolomyces lactucae*	99.47%	Egypt	flower, chamomile	Z.S.M. Soliman unpubl.
KY611834	*Sporobolomyces roseus*	AUMC 11233	*Sporobolomyces lactucae*	99.65%	Egypt	flower, mango (Mangifera indica)	Z.S.M. Soliman unpubl.
MF071283	*Sporobolomyces roseus* (as *Sporidiobolus metaroseus*)	AUMC 11218	*Sporobolomyces lactucae*	99.64%	Egypt	flower of Rosaceae plant	Z.S.M. Soliman unpubl.

## Data Availability

*Sporobolomyces lactucae* cultures were deposited in the USDA-ARS Collection (NRRL) and the Westerdijk Fungal Biodiversity Institute (CBS). Sequence data are available via GenBank (NCBI), and aligned data sets are available on TreeBase (Project number 29521; http://purl.org/phylo/treebase/phylows/study/TB2:S29521, accessed on 27 January 2022).

## Supplementary Materials

The following supporting information can be downloaded at the following website: https://www.mdpi.com/article/10.3390/jof8030302/s1, Figure S1: Maximum likelihood tree of *Sporobolomyces* s.s. species using the cyt-b locus, Figure S2: Maximum likelihood tree of *Sporobolomyces* s.s. species using the ITS locus, Figure S3: Maximum likelihood tree of *Sporobolomyces* s.s. species using the LSU D1/D2 locus, Figure S4: Maximum likelihood tree of *Sporobolomyces* s.s. species using the tef-1 locus, Table S1: Isolates that were examined in the description of the new species *Sporobolomyces lactucae*. All HU strains were isolated via lettuce leaf homogenization and plated. All MCA strains were isolated via spore drop method, noted as SDC (spore drop culture) in the table below [27]. Holotype is denoted in bold, Table S2: Carbon and nitrogen assimilations and high osmotic pressure tests for all *Sporobolomyces* s.s. species included in phylogenetic analysis in this manuscript. Weak = w; delayed = d; variable = v, and Table S3: Lettuce origins of samples from which *S. lactucae* was isolated based on publicly available data. Lettuce samples were cross-referenced with brand and retailer. Suppliers of identified brands and/or retailers were determined via a public search engine.
