# Supplementary material for: Sporobolomyces lactucae sp. nov. (Pucciniomycotina, Microbotryomycetes, Sporidiobolales): An Abundant Component of Romaine Lettuce Phylloplanes
Source: J Fungi (Basel). 2022 Mar 16;8(3):302. doi: 10.3390/jof8030302 (PMC8951336; doi:10.3390/jof8030302)
Supplement: Supplementary file 1 [file jof-08-00302-s001.zip › jof-1595102-Supplementary Figures.pdf]

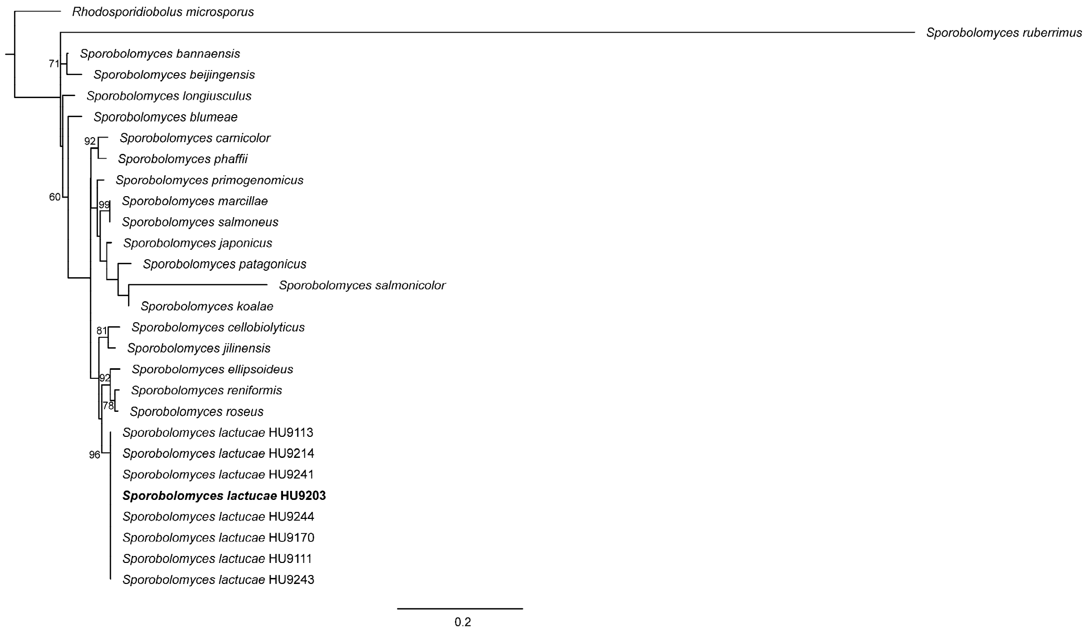

Supplementary Figure S1. Maximum likelihood tree of *Sporobolomyces* s.s. species using the cyt-b locus.

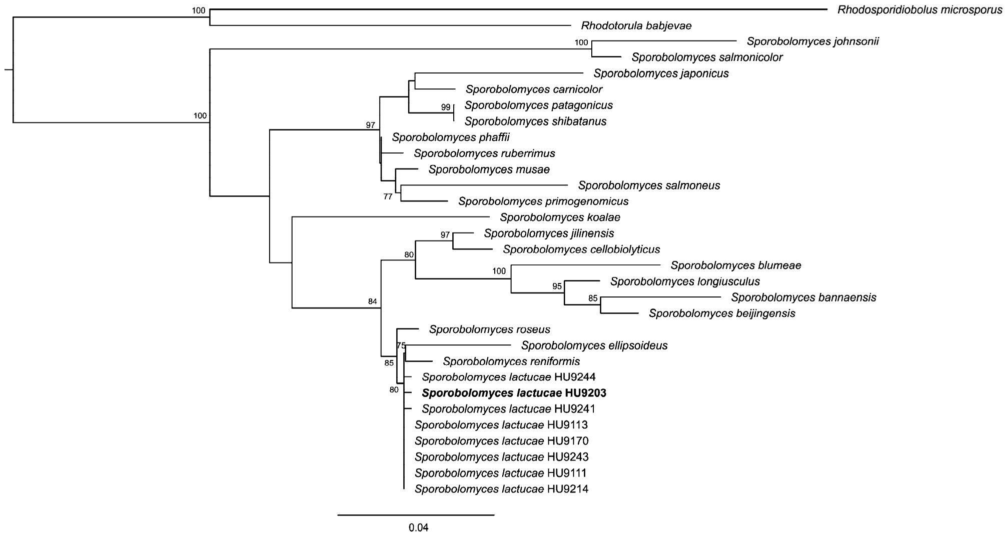

Supplementary Figure S2. Maximum likelihood tree of *Sporobolomyces* s.s. species using the ITS locus.

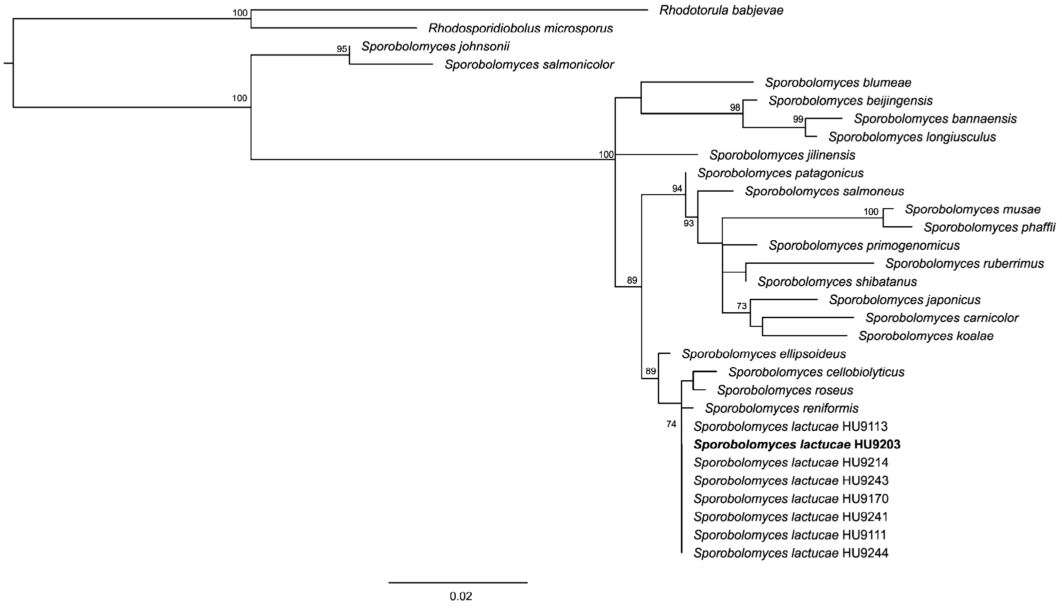

Supplementary Figure S3. Maximum likelihood tree of *Sporobolomyces* s.s. species using the LSU D1/D2 locus.

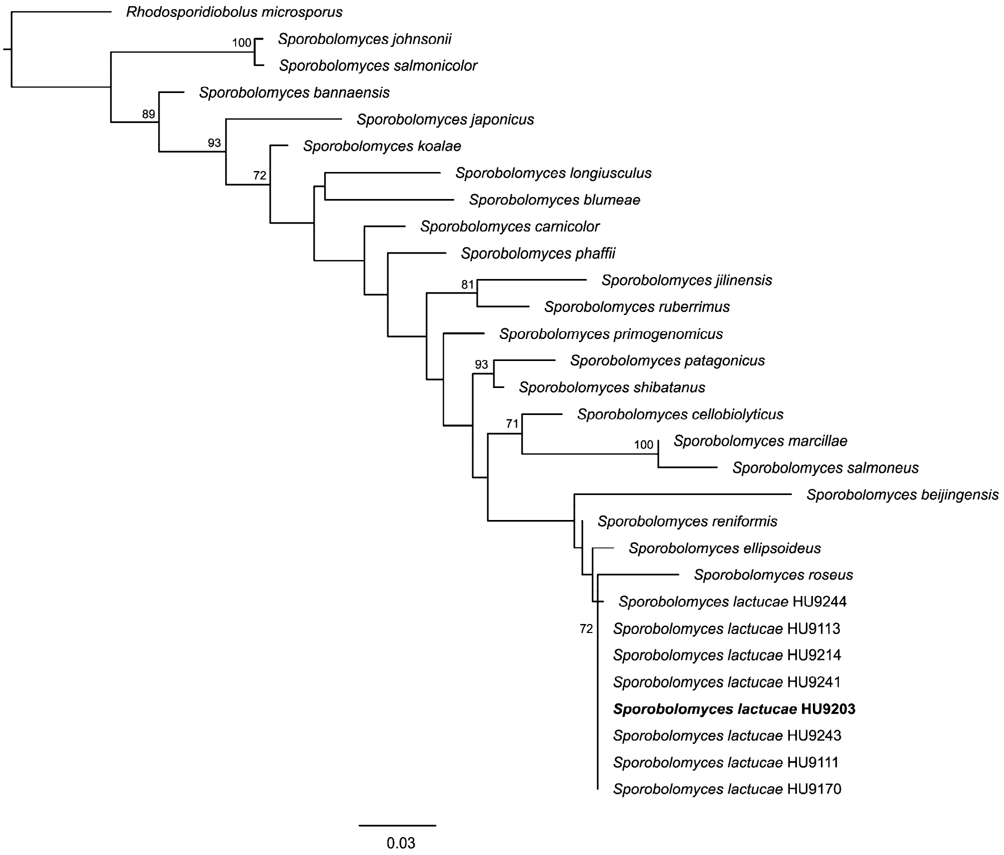

Supplementary Figure S4. Maximum likelihood tree of *Sporobolomyces* s.s. species using the tef-1 locus.
