# Supplementary material for: Sporobolomyces lactucae sp. nov. (Pucciniomycotina, Microbotryomycetes, Sporidiobolales): An Abundant Component of Romaine Lettuce Phylloplanes
Source: J Fungi (Basel). 2022 Mar 16;8(3):302. doi: 10.3390/jof8030302 (PMC8951336; doi:10.3390/jof8030302)
Supplement: Supplementary file 1 [file jof-08-00302-s001.zip › jof-1595102-Supplementary Table S1.pdf]

Supplementary Table S1 Isolates that were examined in the description of the new species Sporobolomyces lactucae. All HU strains were isolated via lettuce leaf homogenization and plated. All MCA strains were isolated via spore drop method, noted as SDC (spore drop

**Isolate**	**Epithet**	**Isolation**	**Plant host**	**Substrate**	**Locality (State)**	**Locality (City)**	**Date Collected**	**ITS**	**LSU**	**SSU**	***tef1***	***cytb***
HU 9007	*Sporobolomyces lactucae*	direct	Asteraceae; *Lactuca sativa*	total leaf	Indiana	Lafayette	2016 Apr 30	MG470871	-	-	-	-
HU 9020	*Sporobolomyces lactucae*	direct	Asteraceae; *Lactuca sativa*	total leaf	Indiana	Lafayette	2016 Apr 30	MG470872	-	-	-	-
HU 9031	*Sporobolomyces lactucae*	direct	Asteraceae; *Lactuca sativa*	total leaf	Indiana	Lafayette	2016 Apr 30	MG470873	-	-	-	-
HU 9034	*Sporobolomyces lactucae*	direct	Asteraceae; *Lactuca sativa*	total leaf	Indiana	Lafayette	2016 Apr 30	MG470875	-	-	-	-
HU 9035	*Sporobolomyces lactucae*	direct	Asteraceae; *Lactuca sativa*	total leaf	Indiana	Lafayette	2016 Apr 30	MG470876	-	-	-	-
HU 9036	*Sporobolomyces lactucae*	direct	Asteraceae; *Lactuca sativa*	total leaf	Indiana	Lafayette	2016 Apr 30	MG470877	-	-	-	-
HU 9047	*Sporobolomyces lactucae*	direct	Asteraceae; *Lactuca sativa*	total leaf	Indiana	Lafayette	2016 May 02	MG470878	-	-	-	-
HU 9049	*Sporobolomyces lactucae*	direct	Asteraceae; *Lactuca sativa*	total leaf	Indiana	Lafayette	2016 May 02	MG470879	-	-	-	-
HU 9062	*Sporobolomyces lactucae*	direct	Asteraceae; *Lactuca sativa*	total leaf	Indiana	Lafayette	2016 May 06	MG470880	-	-	-	-
HU 9065	*Sporobolomyces lactucae*	direct	Asteraceae; *Lactuca sativa*	total leaf	Indiana	Lafayette	2016 May 06	MG470881	-	-	-	-
HU 9074	*Sporobolomyces lactucae*	direct	Asteraceae; *Lactuca sativa*	total leaf	Indiana	Lafayette	2016 May 06	MG470882	-	-	-	-
HU 9076	*Sporobolomyces*	direct	Asteraceae; *Lactuca sativa*	total leaf	Indiana	Lafayette	2016 May 06	MG470883	-	-	-	-

	lactucae											
HU 9091	*Sporobolomyces lactucae*	direct	Asteraceae; *Lactuca sativa*	total leaf	Indiana	Lafayette	2016 May 02	MG470884	-	-	-	-
HU 9092	*Sporobolomyces lactucae*	direct	Asteraceae; *Lactuca sativa*	total leaf	Indiana	Lafayette	2016 May 06	MG470885	-	-	-	-
HU 9100	*Sporobolomyces lactucae*	direct	Asteraceae; *Lactuca sativa*	total leaf	Indiana	Lafayette	2016 May 06	MG470886	-	-	-	-
HU 9101	*Sporobolomyces lactucae*	direct	Asteraceae; *Lactuca sativa*	total leaf	Indiana	Lafayette	2016 May 06	MG470887	-	-	-	-
HU 9111	*Sporobolomyces lactucae*	direct	Asteraceae; *Lactuca sativa*	total leaf	Indiana	Lafayette	2016 May 02	MG470888	MG588941	MG588988	MG589076	MG589036
HU 9113	*Sporobolomyces lactucae*	direct	Asteraceae; *Lactuca sativa*	total leaf	Indiana	Lafayette	2016 May 06	MG470889	MG588942	MG588989	MG589077	MG589037
HU 9115	*Sporobolomyces lactucae*	direct	Asteraceae; *Lactuca sativa*	total leaf	Indiana	Lafayette	2016 May 06	MG470890	-	-	-	-
HU 9116	*Sporobolomyces lactucae*	direct	Asteraceae; *Lactuca sativa*	total leaf	Indiana	Lafayette	2016 May 06	MG470891	-	-	-	-
HU 9128	*Sporobolomyces lactucae*	direct	Asteraceae; *Lactuca sativa*	total leaf	Indiana	Lafayette	2016 May 06	MG470892	-	-	-	-
HU 9129	*Sporobolomyces lactucae*	direct	Asteraceae; *Lactuca sativa*	total leaf	Indiana	Lafayette	2016 May 06	MG470893	-	-	-	-
HU 9133	*Sporobolomyces lactucae*	direct	Asteraceae; *Lactuca sativa*	total leaf	Indiana	Lafayette	2016 May 06	MG470894	-	-	-	-
HU 9137	*Sporobolomyces lactucae*	direct	Asteraceae; *Lactuca sativa*	total leaf	Indiana	Lafayette	2016 May 06	MG470895	-	-	-	-
HU 9143	*Sporobolomyces lactucae*	direct	Asteraceae; *Lactuca sativa*	total leaf	Indiana	Lafayette	2016 May 06	MG470897	-	-	-	-

HU 9146	*Sporobolomyces lactucae*	direct	Asteraceae; *Lactuca sativa*	total leaf	Indiana	Lafayette	2016 May 06	MG470898	MG588943	MG588990	MG589078	MG589038
HU 9148	*Sporobolomyces lactucae*	direct	Asteraceae; *Lactuca sativa*	total leaf	Indiana	Lafayette	2016 May 06	MG470899	-	-	-	-
HU 9152	*Sporobolomyces lactucae*	direct	Asteraceae; *Lactuca sativa*	total leaf	Indiana	West Lafayette	2016 May 08	MG470892	-	-	-	-
HU 9155	*Sporobolomyces lactucae*	direct	Asteraceae; *Lactuca sativa*	total leaf	Indiana	West Lafayette	2016 May 08	MG470901	-	-	-	-
HU 9163	*Sporobolomyces lactucae*	direct	Asteraceae; *Lactuca sativa*	total leaf	Indiana	West Lafayette	2016 May 08	MG470902	-	-	-	-
HU 9170	*Sporobolomyces lactucae*	direct	Asteraceae; *Lactuca sativa*	total leaf	Indiana	West Lafayette	2016 May 08	MG470903	MG588944	MG588991	MG589079	MG589039
HU 9175	*Sporobolomyces lactucae*	direct	Asteraceae; *Lactuca sativa*	total leaf	Indiana	West Lafayette	2016 May 08	MG470904	MG588945	MG588992	MG589080	-
HU 9180	*Sporobolomyces lactucae*	direct	Asteraceae; *Lactuca sativa*	total leaf	Illinois	Champaign	2016 May 09	MG470905	-	-	-	-
HU 9185	*Sporobolomyces lactucae*	direct	Asteraceae; *Lactuca sativa*	total leaf	Illinois	Champaign	2016 May 09	MG470906	-	-	-	-
HU 9186	*Sporobolomyces lactucae*	direct	Asteraceae; *Lactuca sativa*	total leaf	Illinois	Champaign	2016 May 09	MG470907	-	-	-	-
HU 9188	*Sporobolomyces lactucae*	direct	Asteraceae; *Lactuca sativa*	total leaf	Illinois	Champaign	2016 May 09	MG470908	-	-	-	-
HU 9192	*Sporobolomyces lactucae*	direct	Asteraceae; *Lactuca sativa*	total leaf	Illinois	Champaign	2016 May 09	MG470909	-	-	-	-
HU 9197	*Sporobolomyces lactucae*	direct	Asteraceae; *Lactuca sativa*	total leaf	Illinois	Champaign	2016 May 09	MG470910	-	-	-	-

HU 9202	*Sporobolomyces lactucae*	direct	Asteraceae; *Lactuca sativa*	total leaf	Illinois	Champaign	2016 May 09	MG470911	MG588946	MG588993	MG589081	MG589040
**HU 9203**	***Sporobolomyces lactucae***	**direct**	**Asteraceae; *Lactuca sativa***	**total leaf**	Illinois	**Champaign**	**2016 May 09**	**MG470912**	**MG588947**	**MG588994**	**MG589082**	**MG589041**
HU 9204	*Sporobolomyces lactucae*	direct	Asteraceae; *Lactuca sativa*	total leaf	Illinois	Champaign	2016 May 09	MG470913	MG588948	MG588995	MG589083	MG589042
HU 9206	*Sporobolomyces lactucae*	direct	Asteraceae; *Lactuca sativa*	total leaf	Illinois	Champaign	2016 May 09	MG470914	-	-	-	-
HU 9208	*Sporobolomyces lactucae*	direct	Asteraceae; *Lactuca sativa*	total leaf	Illinois	Champaign	2016 May 09	MG470915	-	-	-	-
HU 9213	*Sporobolomyces lactucae*	direct	Asteraceae; *Lactuca sativa*	total leaf	Illinois	Champaign	2016 May 09	MG470916	-	-	-	-
HU 9214	*Sporobolomyces lactucae*	direct	Asteraceae; *Lactuca sativa*	total leaf	Illinois	Champaign	2016 May 09	MG470917	MG588949	MG588996	MG589084	MG589043
HU 9216	*Sporobolomyces lactucae*	direct	Asteraceae; *Lactuca sativa*	total leaf	Illinois	Champaign	2016 May 09	MG470918	-	-	-	-
HU 9233	*Sporobolomyces lactucae*	direct	Asteraceae; *Lactuca sativa*	total leaf	Illinois	Champaign	2016 May 09	-	-	-	-	-
HU 9235	*Sporobolomyces lactucae*	direct	Asteraceae; *Lactuca sativa*	total leaf	Illinois	Champaign	2016 May 09	MG470920	MG588950	MG588997	MG589085	MG589044
HU 9241	*Sporobolomyces lactucae*	direct	Asteraceae; *Lactuca sativa*	total leaf	Illinois	Chicago	2016 May 19	MG470921	MG588951	MG588998	MG589086	MG589045
HU 9243	*Sporobolomyces lactucae*	direct	Asteraceae; *Lactuca sativa*	total leaf	Illinois	Chicago	2016 May 19	MG470922	MG588952	MG588999	MG589087	MG589046
HU 9244	*Sporobolomyces lactucae*	direct	Asteraceae; *Lactuca sativa*	total leaf	Illinois	Chicago	2016 May 19	MG470923	MG588953	MG589000	MG589088	MG589047

HU 9246	*Sporobolomyces lactucae*	direct	Asteraceae; *Lactuca sativa*	total leaf	Illinois	Chicago	2016 May 19	MG470924	-	-	-	-
HU 9249	*Sporobolomyces lactucae*	direct	Asteraceae; *Lactuca sativa*	total leaf	Illinois	Chicago	2016 May 19	MG470925	-	-	-	-
HU 9250	*Sporobolomyces lactucae*	direct	Asteraceae; *Lactuca sativa*	total leaf	Illinois	Chicago	2016 May 19	MG470926	-	-	-	-
HU 9251	*Sporobolomyces lactucae*	direct	Asteraceae; *Lactuca sativa*	total leaf	Illinois	Chicago	2016 May 19	MG470927	-	-	-	-
HU 9255	*Sporobolomyces lactucae*	direct	Asteraceae; *Lactuca sativa*	total leaf	Illinois	Chicago	2016 May 19	MG470928	-	-	-	-
HU 9257	*Sporobolomyces lactucae*	direct	Asteraceae; *Lactuca sativa*	total leaf	Illinois	Chicago	2016 May 19	MG470929	-	-	-	-
HU 9266	*Sporobolomyces lactucae*	direct	Asteraceae; *Lactuca sativa*	total leaf	Illinois	Chicago	2016 May 19	MG470930	-	-	-	-
HU 9268	*Sporobolomyces lactucae*	direct	Asteraceae; *Lactuca sativa*	total leaf	Illinois	Chicago	2016 May 19	MG470931	-	MG589001	-	-
HU 9272	*Sporobolomyces lactucae*	direct	Asteraceae; *Lactuca sativa*	total leaf	Illinois	Chicago	2016 May 19	MG470932	-	-	-	-
HU 9273	*Sporobolomyces lactucae*	direct	Asteraceae; *Lactuca sativa*	total leaf	Illinois	Chicago	2016 May 19	MG470933	-	-	-	-
HU 9279	*Sporobolomyces lactucae*	direct	Asteraceae; *Lactuca sativa*	total leaf	Illinois	Chicago	2016 May 19	MG470934	-	-	-	-
HU 9281	*Sporobolomyces lactucae*	direct	Asteraceae; *Lactuca sativa*	total leaf	Illinois	Chicago	2016 May 19	MG470935	-	-	-	-
HU 9285	*Sporobolomyces lactucae*	direct	Asteraceae; *Lactuca sativa*	total leaf	Illinois	Chicago	2016 May 19	MG470936	-	-	-	-

HU 9286	*Sporobolomyces lactucae*	direct	Asteraceae; *Lactuca sativa*	total leaf	Illinois	Chicago	2016 May 19	MG470937	-	-	MG589089	MG589048
MCA6380	*Sporobolomyces lactucae*	SDC	Rhamnaceae; *Ceanothus arboreus*	phylloplane - diseased leaf - *Septoria blasdalei* MCA 6369	California	Berkeley	2016 Aug 05	MG471074	-	-	-	-
MCA6381	*Sporobolomyces lactucae*	SDC	Rhamnaceae; *Ceanothus arboreus*	phylloplane - diseased leaf - *Septoria blasdalei* MCA 6370	California	Berkeley	2016 Aug 05	MG471075	-	-	-	-
MCA6382	*Sporobolomyces lactucae*	SDC	Rhamnaceae; *Ceanothus arboreus*	phylloplane - healthy leaf	California	Berkeley	2016 Aug 05	MG471076	-	-	-	-
MCA6385	*Sporobolomyces lactucae*	SDC	Plantaginaceae; *Antirrhinum majus*	phylloplane - healthy leaf	California	Berkeley	2016 Aug 05	MG471078	-	-	-	-
MCA6386	*Sporobolomyces lactucae*	SDC	Plantaginaceae; *Antirrhinum majus*	phylloplane - diseased leaf - *Puccinia antirrhini* MCA 6366	California	Berkeley	2016 Aug 05	MG471079	-	-	-	-
MCA6387	*Sporobolomyces lactucae*	SDC	Plantaginaceae; *Antirrhinum majus*	phylloplane - diseased leaf - *Puccinia antirrhini* MCA 6366	California	Berkeley	2016 Aug 05	MG471080	-	-	-	-
MCA6391	*Sporobolomyces lactucae*	SDC	Plantaginaceae; *Antirrhinum majus*	phylloplane - healthy flower	California	Berkeley	2016 Aug 05	MG471082	-	-	-	-
MCA6392	*Sporobolomyces lactucae*	SDC	Plantaginaceae; *Antirrhinum majus*	phylloplane - healthy flower	California	Berkeley	2016 Aug 05	MG471083	-	-	-	-
MCA6393	*Sporobolomyces lactucae*	SDC	Plantaginaceae; *Antirrhinum majus*	phylloplane - healthy flower	California	Berkeley	2016 Aug 05	MG471084	-	-	-	-

MCA6394	*Sporobolomyces lactucae*	SDC	Plantaginaceae; *Antirrhinum majus*	phylloplane - healthy flower	California	Berkeley	2016 Aug 05	MG471085	-	-	-	-
MCA6395	*Sporobolomyces lactucae*	SDC	Liliaceae	phylloplane - necrotic leaf	California	Berkeley	2016 Aug 05	MG471086	-	-	-	-
MCA6396	*Sporobolomyces lactucae*	SDC	Liliaceae	phylloplane - necrotic leaf	California	Berkeley	2016 Aug 05	MG471087	-	-	-	-
MCA6397	*Sporobolomyces lactucae*	SDC	Plantaginaceae; *Antirrhinum majus*	phylloplane - diseased leaf - *Puccinia antirrhini* MCA 6366	California	Berkeley	2016 Aug 05	MG471088	-	-	-	-
MCA6398	*Sporobolomyces lactucae*	SDC	Plantaginaceae; *Antirrhinum majus*	phylloplane - necrotic leaf	California	Berkeley	2016 Aug 05	MG471089	-	-	-	-
MCA6399	*Sporobolomyces lactucae*	SDC	Plantaginaceae; *Antirrhinum majus*	phylloplane - diseased leaf - *Puccinia antirrhini* MCA 6366	California	Berkeley	2016 Aug 05	MG471090	-	-	-	-
MCA6400	*Sporobolomyces lactucae*	SDC	-	phylloplane	California	Berkeley	2016 Aug 05	OK624451	-	-	-	-
MCA6401	*Sporobolomyces lactucae*	SDC	Plantaginaceae; *Antirrhinum majus*	phylloplane - necrotic leaf	California	Berkeley	2016 Aug 05	MG471091	-	-	-	-
MCA6402	*Sporobolomyces lactucae*	SDC	Plantaginaceae; *Antirrhinum majus*	phylloplane - healthy flower	California	Berkeley	2016 Aug 05	MG471092	-	-	-	-
MCA8251	*Sporobolomyces lactucae*	SDC	-	phylloplane	California	Berkeley	2019 Mar	OK623656	-	-	-	-
MCA8252	*Sporobolomyces lactucae*	SDC	-	phylloplane	California	Berkeley	2019 Mar	OK623652	-	-	-	-
MCA8256	*Sporobolomyces*	SDC	-	phylloplane	California	Berkeley	2019 Mar	OK623653	-	-	-	-

	lactucae												
MCA8258	*Sporobolomyces lactucae*	SDC	-		phylloplane	California	Berkeley	2019 Mar	MZ686261	-	-	-	-
MCA8260	*Sporobolomyces lactucae*	SDC	-		phylloplane	California	Berkeley	2019 Mar	OK586496	-	-	-	-
MCA8261	*Sporobolomyces lactucae*	SDC	-		phylloplane	California	Berkeley	2019 Mar	OK623658	-	-	-	-
MCA8283	*Sporobolomyces lactucae*	SDC	-		phylloplane	California	Berkeley	2019 Mar	OK623657	-	-	-	-
MCA8284	*Sporobolomyces lactucae*	SDC	-		phylloplane	California	Berkeley	2019 Mar	OK586497	-	-	-	-
MCA8285	*Sporobolomyces lactucae*	SDC	-		phylloplane	California	Berkeley	2019 Mar	OK586495	-	-	-	-
