# Supplementary material for: Sporobolomyces lactucae sp. nov. (Pucciniomycotina, Microbotryomycetes, Sporidiobolales): An Abundant Component of Romaine Lettuce Phylloplanes
Source: J Fungi (Basel). 2022 Mar 16;8(3):302. doi: 10.3390/jof8030302 (PMC8951336; doi:10.3390/jof8030302)
Supplement: Supplementary file 1 [file jof-08-00302-s001.zip › jof-1595102-Supplementary Table S2.pdf]

Supplementary Table S2. Carbon & nitrogen assimilations and high osmotic pressure tests for all Sporobolomyces s.s. species included in phylogenetic analysis in this manuscript. Weak = w; delayed = d; variable = v.

	Species	*Sporobolomyces lactucae*	*S. bannaensis*	*S. beijingensis*	*S. blumeae*	*S. carnicolor*	*S. japonicus*	*S. jilinensis*	*S. johnsonii*	*S. koalae*	*S. longiusculus*	*S. marcillae*	*S. patagonicus*	*S. phaffii*	*S. roseus*	*S. ruberrimus*	*S. salmoneus*	*S. salmonicolor*
	Type Strain	HU9203	AS2.2285	AS2.2365	JCM 10212	JCM 3766	AS2.2192	AS2.2301	AS2.1927	CBS 10914	CBS 9655	JCM 6883	CBS 9657	AS2.2137	AS2.1948	CBS 7550	AS2.2137	JCM 1841
	Reference	This paper	Zhao et. al. 2003	Wang and Bai 2004	Kurtzman, Fell & Boekhout 2011a	Kurtzman, Fell & Boekhout 2011a	Kurtzman, Fell & Boekhout 2011a; Iizuka and Goto 1965	Wang and Bai 2004	Kurtzman, Fell & Boekhout 2011a	Satoh and Makimura 2008	Libkind et. al. 2005	Santa Maria 1958	Libkind et. al. 2005	Bai et. al. 2002	Kurtzman, Fell & Boekhout 2011a	Fell et. al. 2002	Kurtzman, Fell & Boekhout 2011a	Kurtzman, Fell & Boekhout 2011a
CARBON	Glucose	+	+	+	+	+	+	+	+	+	+	+	+	+	+	+	+	+
	Galactose	+ (w, d)	+	-	-	d	+	+	-	-	+	+	d	-	+	d	v	-
	Sucrose	+	+	+	+	+	+	+	+	+	+	+	+	+	+	+	+	+
	Maltose	+	+	+	+	+	+	+	+	+	+	+	v	+	+	+	+	+
	Cellobiose	+ (w, d)	+	+	d	+	+	-	+	+	+	n/a	v	+	+	+	+	+
	Lactose	-	-	-	-	-	-	-	-	-	-	+	-	-	-	-	-	-
	Inulin	-	+	-	-	- (w)	-	-	-	+ (w)	-	n/a	-	-	-	-	-	-
	Soluble starch	+ (w)	+	+	+	- (w)	+	+	+	+	+	n/a	+	+	+	-	- (w)	-
	myo-Inositol	-	-	-	-	-	-	-	-	-	-	n/a	-	-	-	-	-	-
	Glucono-1,5-lactone	+ (w)	n/a	n/a	w	+	+	n/a	n/a	n/a	n/a	n/a	+	+	n/a	-	- (w)	n/a
	Glucuronate	+	n/a	n/a	n/a	-	-	n/a	-	n/a	-	n/a	-	-	-	-	n/a	-
	Galacturonic Acid	- (w)	n/a	n/a	n/a	-	-	n/a	n/a	n/a	n/a	n/a	n/a	-	-	-	-	n/a
	Lactate	-	- (as lactic acid)	- (as lactic acid)	-	-	w	- (as DL-lactic acid)	v	- (as lactic acid)	-	n/a	-	- (as lactic acid)	+	-	-	v
	Citrate	-	- (as citric acid)	- (as citric acid)	-	+	+	- (as citric acid)	+	- (as citric acid)	-	n/a	-	+ (as citric acid)	+	- (w)	v	+
	Methanol	-	-	-	-	-	-	-	-	-	-	n/a	n/a	n/a	-	- (w)	-	-
	Ethanol	- (w)	+	+	-	+	+	+	+	-	+	+	-	- (w)	+	+	+	+
	Propane-1,2-diol	- (w)	n/a	n/a	n/a	n/a	n/a	n/a	n/a	n/a	n/a	n/a	n/a	n/a	n/a	-(w)	n/a	n/a
NITROGEN	K Nitrate	+	+	-	+	-	-	+	+	+	-	-	+	+	+	+	v	+
	Na Nitrite	+	+	-	-	-	-	+	+	-	-	n/a	-	+	+	+	v	+
	Ethylamine	- (w)	-	-	-	-	+	-	n/a	-	-	n/a	+	v	n/a	-	v	n/a
	L-lysine	+ (w)	-	+	+	+	+	+	n/a	-	n/a	n/a	v	+ (w)	n/a	- (w)	v	n/a
	Cadaverine	- (w)	-	-	w	+	+	+	n/a	-	-	n/a	v	- (w)	n/a	+	v	n/a
	Creatine	- (w)	n/a	n/a	n/a	n/a	n/a	n/a	n/a	n/a	n/a	n/a	n/a	n/a	n/a	-	n/a	n/a
	Creatinine	- (w)	n/a	n/a	n/a	n/a	n/a	n/a	n/a	n/a	n/a	n/a	n/a	n/a	n/a	-	n/a	n/a
	D-glucosamine	+	-	-	-	-	-	-	-	-	+	n/a	-	n/a	-	-	-	-
	Imidazole	-	n/a	n/a	n/a	n/a	n/a	n/a	n/a	n/a	-	n/a	n/a	n/a	n/a	-	n/a	n/a
	D-tryptophan	- (w)	n/a	n/a	n/a	n/a	n/a	n/a	n/a	n/a	-	n/a	n/a	n/a	n/a	+ (w)	n/a	n/a
HIG	10% (w/v) NaCl	- (w)	n/a	n/a	n/a	n/a	n/a	n/a	n/a	n/a	n/a	n/a	n/a	n/a	n/a	n/a	n/a	n/a

16% (w/v) NaCl	- (w)	n/a	n/a	n/a	n/a	n/a	n/a	n/a	n/a	n/a	n/a	n/a	n/a	n/a	n/a	n/a	n/a
50% (w/v) Glucose	+	n/a	+	n/a	+	- (w)	-	n/a	-	n/a	n/a	n/a	n/a	v	n/a	- (w)	-
60% (w/v) Glucose	+	n/a	n/a	n/a	n/a	n/a	n/a	n/a	n/a	n/a	n/a	n/a	n/a	n/a	n/a	n/a	n/a
