# Supplementary material for: Sporobolomyces lactucae sp. nov. (Pucciniomycotina, Microbotryomycetes, Sporidiobolales): An Abundant Component of Romaine Lettuce Phylloplanes
Source: J Fungi (Basel). 2022 Mar 16;8(3):302. doi: 10.3390/jof8030302 (PMC8951336; doi:10.3390/jof8030302)
Supplement: Supplementary file 1 [file jof-08-00302-s001.zip › jof-1595102-Supplementary Table S3.pdf]

Supplementary Table S3 Lettuce origins of samples from which S. lactucae was isolated based on publicly available data. Lettuce samples were cross-referenced with brand and retailer. Suppliers of identified brands and/or retailers were determined via public search.

ISOLATE	RETAILER	PRODUCER	REGION	STATE	NOTES
**HU 9007**	Walmart	Church Brothers Farms	Yuma	AZ	May - Oct production in Salinas, CA
**HU 9020**	Fresh Thyme	–	–	–	–
**HU 9031**	Fresh Thyme	–	–	–	–
**HU 9034**	Fresh Thyme	–	–	–	–
**HU 9035**	Fresh Thyme	–	–	–	–
**HU 9036**	Fresh Thyme	–	–	–	–
**HU 9047**	Target	Church Brothers Farms	Yuma	AZ	May - Oct production in Salinas, CA
**HU 9049**	Target	Church Brothers Farms	Yuma	AZ	May - Oct production in Salinas, CA
**HU 9062**	Payless	Josie's	Soledad	CA	Organic
**HU 9065**	Payless	Josie's	Soledad	CA	Organic
**HU 9074**	Payless	–	–	–	–
**HU 9076**	Payless	–	–	–	–
**HU 9091**	Target	Church Brothers Farms	Yuma	AZ	May - Oct production in Salinas, CA
**HU 9092**	Payless	–	–	–	–
**HU 9100**	Payless	–	–	–	–
**HU 9101**	Payless	–	–	–	–
**HU 9111**	Target	Church Brothers Farms	Yuma	AZ	May - Oct production in Salinas, CA
**HU 9113**	Payless	Josie's	Soledad	CA	Organic
**HU 9115**	Payless	Josie's	Soledad	CA	Organic
**HU 9116**	Payless	Josie's	Soledad	CA	Organic
**HU 9128**	Payless	Josie's	Soledad	CA	Organic
**HU 9129**	Payless	Josie's	Soledad	CA	Organic
**HU 9133**	Payless	–	–	–	–
**HU 9137**	Payless	–	–	–	–
**HU 9143**	Payless	–	–	–	–
**HU 9146**	Payless	–	–	–	–
**HU 9148**	Payless	–	–	–	–
**HU 9152**	Meijer	Ocean Mist	Coachella/Salinas	CA	–
**HU 9155**	Meijer	Ocean Mist	Coachella/Salinas	CA	–
**HU 9163**	Meijer	Ocean Mist	Coachella/Salinas	CA	–
**HU 9170**	Meijer	Ocean Mist	Coachella/Salinas	CA	–
**HU 9175**	Meijer	Ocean Mist	Coachella/Salinas	CA	–
**HU 9180**	Meijer	Ocean Mist	Coachella/Salinas	CA	–
**HU 9185**	Meijer	Ocean Mist	Coachella/Salinas	CA	–
**HU 9186**	Meijer	Ocean Mist	Coachella/Salinas	CA	–
**HU 9188**	Meijer	Ocean Mist	Coachella/Salinas	CA	–
**HU 9192**	Meijer	Ocean Mist	Coachella/Salinas	CA	–
**HU 9197**	Meijer	Ocean Mist	Coachella/Salinas	CA	–
**HU 9202**	Walmart	Tanimura and Antle	Salinas	CA	Nov - Mar production in Yuma, AZ
**HU 9203**	Walmart	Tanimura and Antle	Salinas	CA	Nov - Mar production in Yuma, AZ

**HU 9204**	Walmart	Tanimura and Antle	Salinas	CA	Nov - Mar production in Yuma, AZ
**HU 9206**	Walmart	Tanimura and Antle	Salinas	CA	Nov - Mar production in Yuma, AZ
**HU 9208**	Walmart	Tanimura and Antle	Salinas	CA	Nov - Mar production in Yuma, AZ
**HU 9213**	Walmart	Tanimura and Antle	Salinas	CA	Nov - Mar production in Yuma, AZ
**HU 9214**	Walmart	Tanimura and Antle	Salinas	CA	Nov - Mar production in Yuma, AZ
**HU 9216**	Walmart	Tanimura and Antle	Salinas	CA	Nov - Mar production in Yuma, AZ
**HU 9233**	Walmart	Tanimura and Antle	Salinas	CA	Nov - Mar production in Yuma, AZ
**HU 9235**	Walmart	Tanimura and Antle	Salinas	CA	Nov - Mar production in Yuma, AZ
**HU 9241**	Walmart	–	–	–	–
**HU 9243**	Walmart	–	–	–	–
**HU 9244**	Walmart	Tanimura and Antle	Salinas	CA	Nov - Mar production in Yuma, AZ
**HU 9246**	Walmart	Tanimura and Antle	Salinas	CA	Nov - Mar production in Yuma, AZ
**HU 9249**	Walmart	Tanimura and Antle	Salinas	CA	Nov - Mar production in Yuma, AZ
**HU 9250**	Walmart	Tanimura and Antle	Salinas	CA	Nov - Mar production in Yuma, AZ
**HU 9251**	Walmart	Tanimura and Antle	Salinas	CA	Nov - Mar production in Yuma, AZ
**HU 9255**	Walmart	–	–	–	–
**HU 9257**	Walmart	–	–	–	–
**HU 9266**	Walmart	–	–	–	–
**HU 9268**	Walmart	–	–	–	–
**HU 9272**	Walmart	–	–	–	–
**HU 9273**	Walmart	–	–	–	–
**HU 9279**	Walmart	Tanimura and Antle	Salinas	CA	Nov - Mar production in Yuma, AZ
**HU 9281**	Walmart	Tanimura and Antle	Salinas	CA	Nov - Mar production in Yuma, AZ
**HU 9285**	Walmart	Tanimura and Antle	Salinas	CA	Nov - Mar production in Yuma, AZ
**HU 9286**	Walmart	Tanimura and Antle	Salinas	CA	Nov - Mar production in Yuma, AZ
